# Optimizing Dermal Filler for Chin and Jawline Definition as an Advanced Approach for Natural Results: A Prospective Case Series With Ultrasonographic and 3D Facial Imaging Evaluation

**DOI:** 10.7759/cureus.99477

**Published:** 2025-12-17

**Authors:** Allan Alcantara, Érika Vassoler Guerrero Puccia, Caio Perrella de Rezende, Jun Ho Kim, Renata Viana

**Affiliations:** 1 Dentistry, Instituto Nacional de Reabilitação Orofacial (INRO), São Paulo, BRA; 2 Stomatology, Universidade de São Paulo, São Paulo, BRA; 3 Surgery, Private Practice, São Paulo, BRA

**Keywords:** aesthetic medicine, chin, dermal filler, jawline, non-surgical contouring

## Abstract

Non-surgical mandibular contouring has gained attention as a safe and effective approach to improve jawline aesthetics and restore lower facial definition. This study aimed to assess the efficacy, safety, and patient satisfaction associated with an anatomically guided hyaluronic acid (HA) filler technique designed to enhance chin projection and mandibular contour using minimal product volume. Ten healthy patients aged 25-45 years underwent individualized treatments guided by facial proportions and anatomical landmarks. A maximum of 6 mL of high elastic modulus (G′) HA filler was administered per patient. Three-dimensional photographic documentation and ultrasound imaging were used to guide applications and monitor outcomes. The results showed consistent enhancement in lower face definition across all patients, with a 98% satisfaction rate and no major adverse events reported. These findings support the use of this anatomically guided protocol as a reproducible and minimally invasive option for lower facial rejuvenation, offering effective aesthetic improvements while maintaining a favorable safety profile.

## Introduction

Facial harmony is a fundamental principle in aesthetic medicine, with the lower third of the face, encompassing the jawline and chin, playing a pivotal role in defining overall attractiveness, youthfulness, and gender-specific characteristics. A well-contoured mandibular region contributes to the perception of balance and symmetry, influencing both frontal and lateral facial views. Changes in this area, whether from genetic factors, aging, or weight fluctuations, can disrupt facial proportions and lead individuals to seek corrective procedures [1-3]. 

The demand for non-surgical rejuvenation has led to the prominence of injectable fillers, particularly hyaluronic acid (HA) derivatives, as a preferred modality for enhancing the mandibular contour. HA is a hydrophilic glycosaminoglycan that forms a three-dimensional (3D) gel matrix in the extracellular space, attracting and retaining water to increase soft tissue volume and mechanical support [4]. These fillers offer the advantages of minimal invasiveness, immediate results, and a favorable safety profile. High elastic modulus (G′) HA fillers, characterized by their robust lifting capacity and cohesivity, are especially suited for the structural augmentation of the jawline and chin, providing durable and natural-looking outcomes [4-6].

Evidence supporting the effectiveness of HA fillers continues to grow. Rauso et al. emphasized the value of rheologically appropriate fillers and multilayer techniques in achieving durable results in jawline enhancement [7]. Similarly, Moradi et al. and Vanaman Wilson et al. demonstrated sustained aesthetic improvement and high patient satisfaction through individualized lower face treatment protocols [5,6].

Due to the anatomical complexity of the mandibular region, it is critical to acknowledge important anatomical facial structures of the area to be treated, such as the facial artery and vein, mental foramen, marginal mandibular nerve, and parotid gland and duct, in order to minimize risks and optimize results [8,9].

In addition to anatomical precision, aesthetic principles such as the golden ratio and cephalometric analysis guide the customization of treatment plans. Evaluating the proportions between the bizygomatic and bigonial widths, as well as the chin's projection relative to other facial landmarks, allows individualized approaches that respect each patient's unique facial architecture and aesthetic goals [10,11].

Advanced imaging technologies, including 3D photography and digital cephalometric analysis, further enhance the ability to plan and execute contouring procedures. These tools, when available, facilitate the assessment of facial symmetry, volume distribution, and treatment planning, as well as the quantitative evaluation of facial volumetric changes and treatment outcomes [11,12].

This article presents a comprehensive approach to non-surgical mandibular enhancement using HA fillers, emphasizing individualized treatment planning, anatomical safety, and aesthetic harmony. By limiting the total filler amount, the study demonstrates that effective contouring can be achieved with low product volumes when anatomical planes and aesthetic principles are carefully respected. The objective of this study was to evaluate the efficacy, safety, and patient satisfaction of an anatomically guided HA filler protocol for chin and jawline enhancement, with particular focus on improvements in chin projection and mandibular contour.

## Materials and methods

A prospective case series was conducted at Instituto Nacional de Reabilitação Orofacial (INRO), São Paulo, Brazil, on 10 healthy patients aged between 25 and 45 years. Lower face enhancement was performed using a high G’ dermal filler, UP Contour (by Ilikia, CGBio, Seoul, South Korea).

Assessment and follow-up images were performed with standardized photography and 3D imaging (QuantifiCare®, Biot, France). Treatments were planned based on cephalometric principles and overall facial proportions. Cephalometric ratios and golden ratio-based assessments were used qualitatively to guide individualized planning, not as standardized quantitative measures.

Ultrasound imaging (EVUS 5, Saevo®, Ribeirão Preto, Brazil, with a high-frequency linear hockey-stick transducer set at 11 MHz) was performed during the procedure to enhance safety, ensuring the correct injection plane and filler placement, and was repeated at follow-up to evaluate filler integration and monitor adverse events. A customized probe-matching template was used to standardize transducer positioning across visits. Three anatomical landmarks were routinely evaluated: the mandibular border (1 cm anterior to the mandibular angle, with the probe oriented longitudinally and parallel to the bone), the menton midline (centered over the chin to assess soft tissue thickness relevant to pogonion projection), and the mandibular notch region (approximately 1 cm posterior to the oral commissure, with the probe aligned parallel to the mandibular border). This standardized protocol allowed the reliable comparison of tissue thickness and filler behavior between pre- and post-treatment scans.

The primary outcome was defined as improvement in chin projection and mandibular contour between baseline and post-treatment assessments (up to 90 days), documented by standardized clinical photography and, when available, ultrasound and 3D imaging. Secondary descriptive outcomes included ultrasound measurements (soft tissue thickness), 3D volumetric changes, patient-reported satisfaction, and adverse events.

Anatomical considerations for a safe procedure

**Figure 1:**
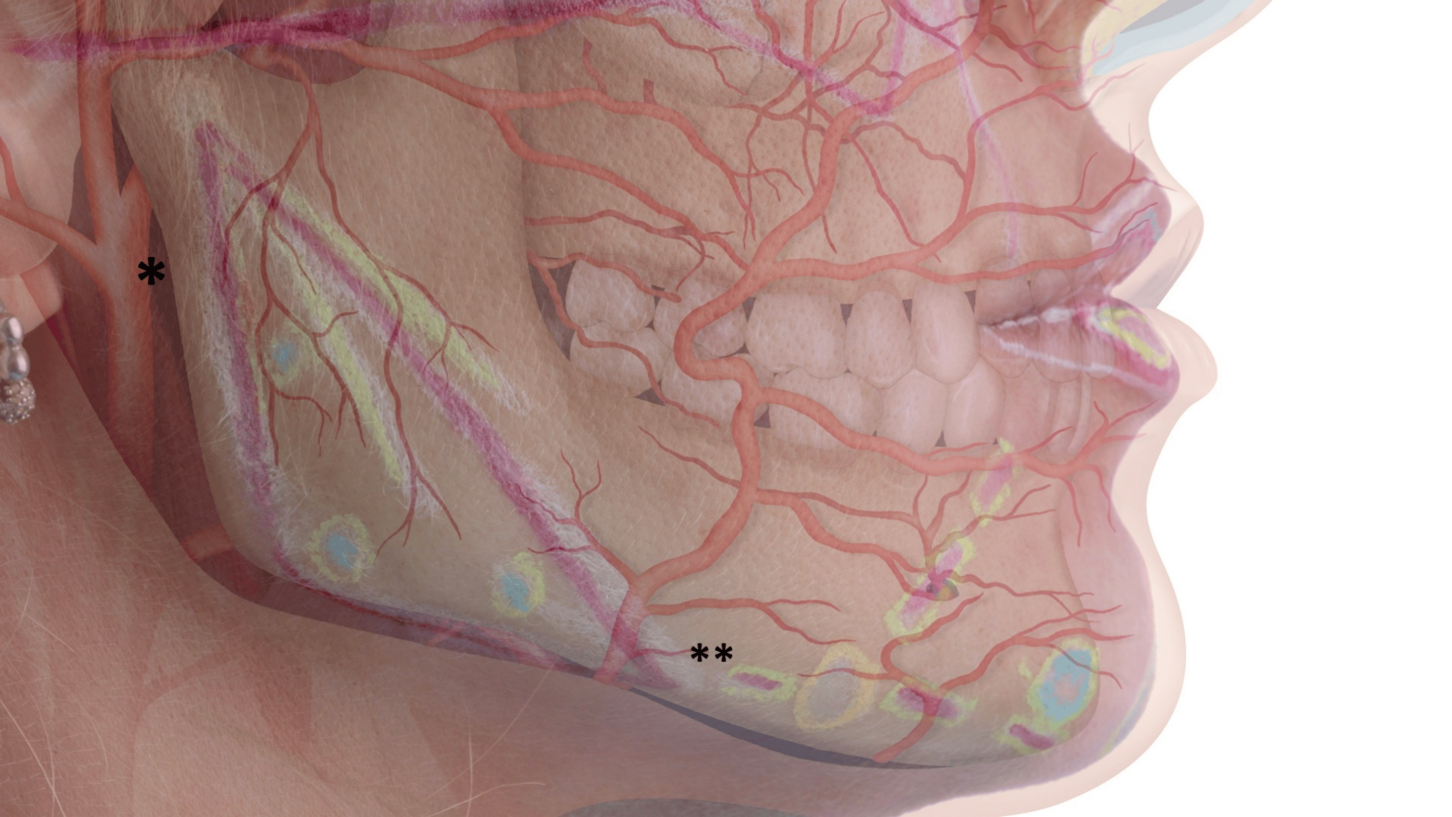
Key vascular structures in the mandibular region *: superficial temporal artery located in the most superficial plane; **: facial artery located in the deep plane

The mandibular region is a complex anatomical area requiring a comprehensive understanding of its vascular, neural, and glandular structures to ensure safe and effective filler application [3,13]. Key anatomical structures (Figure 1) requiring careful attention include the facial artery and vein, the mental foramen and mental nerve, the marginal mandibular branch of the facial nerve, the parotid gland and parotid duct, the superficial temporal artery, and the anterior digastric muscle and submental space.

The facial artery, a branch of the external carotid artery, crosses the mandibular border at the antegonial notch and ascends obliquely across the face. Its course is variable and may be superficial in some individuals, increasing the risk of intravascular injection, hematomas, or tissue necrosis if not identified and avoided. The mental foramen and mental nerve, located near the premolars, transmit the mental nerve and vessels, and inadvertent injections into or near this foramen may cause neuropraxia, leading to temporary or permanent paresthesia of the lower lip and chin. The marginal mandibular branch of the facial nerve travels superficially over the mandibular border, particularly noticeable in patients with age-related subcutaneous fat loss, and trauma to this nerve may cause asymmetric mouth movement or lower lip weakness [13-15].

Filler placement near the parotid gland and parotid duct may encroach upon these structures, particularly if landmarks are poorly identified, and product deposition in this area may cause localized inflammation or parotiditis. Though the superficial temporal artery is not within the mandibular body, its proximity anterior to the tragus (approximately 0.7 cm anterior to the tragus), especially during posterior mandibular injections, necessitates caution, particularly during high-volume contouring near the angle. Overcorrection or excessive filler volume in the region of the anterior digastric muscle and submental space may disrupt the natural submental contour and affect dynamic movements of the lower face.

Safe clinical practice requires anatomical knowledge, application of the product on appropriate injection planes (deep vs. superficial), routine needle aspiration, use of cannulas in high-risk zones, and the incorporation of ultrasound guidance whenever possible to enhance safety and minimize complications.

Treatment planning and injection technique

Treatment planning was systematically structured to allow individualized yet reproducible execution.

Facial Assessment and Proportional Analysis

Assessment was personalized, considering patient-specific attributes such as age, gender, ethnicity, bone structure, and fat distribution. The planning integrated facial analysis, cephalometric ratios, and the principles of the golden ratio (1:1.6) to ensure ideal proportions. Key anatomical landmarks included the pogonion (the most prominent point of the chin), the gonion (the angle of the mandible), the menton (the lowest point of the chin), and the subnasale (the junction of the nose and upper lip).

Assessment parameters included a clinical evaluation of overall facial harmony, considering facial ratios with focus on the visual relationship between bizygomatic and bigonial width, gonial angle, chin morphology, and jawline contour. The physician also identified regions of asymmetry or volume deficit by analyzing the jowl region, observing sagging or loss of definition in the prejowl sulcus, and evaluating subcutaneous fat distribution to guide filler depth and plane selection.

Male Approach

Male aesthetic planning considered an angular lower face, with the bigonial width equal to or slightly greater than the bizygomatic width and a distinctly square, prominent chin [16]. These analyses served as reference patterns and were individually adapted according to each patient's goals, preferences, and personal perceptions of more feminine or masculine characteristics, regardless of gender identity. Facial shape and gonial angle aimed for a defined and square configuration between 90° and 110°, with sharper contours and a broader bigonial width. The jawline contour was enhanced through linear filler injections along the mandibular border to reinforce definition. Chin morphology focused on achieving a wider and squarer contour, referenced to the oral commissures, to provide balanced and natural-looking masculinity.

**Figure 2:**
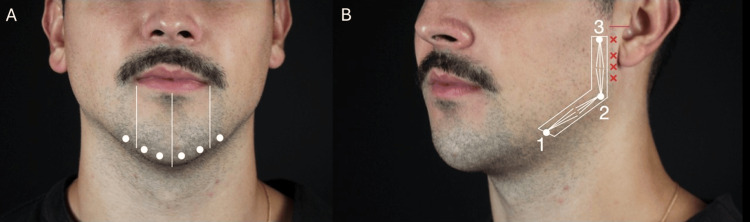
Clinical markings for chin and mandibular angle enhancement used in male patients. (A) Chin filler application showing the delimitation of the treatment area and injection points (4, 6, or 8 points). (B) Mandibular angle marking highlighting the safety zone near the tragus and the entry points (1, 2, and 3)

Chin marking and injection technique (male): Chin marking employed two vertical lines aligned with the oral commissures and a central midline respecting the lower lip projection to ensure symmetry (Figure 2). Typically, 4-8 supraperiosteal injection points were used.

Bolus injections of 0.25 mL HA per point were placed deep supraperiosteally, maximizing projection due to the filler's high G' properties. An aspiration protocol was systematically applied for all needle-based deep supraperiosteal bolus injections as an adjunctive safety measure to minimize the risk of intravascular placement. Aspiration was performed with an "empty syringe" (without preloaded filler) before product injection, with a minimum plunger retraction of approximately 0.2 mL maintained for at least 10 seconds at each injection point, even in traditionally lower-risk regions such as the chin, mandibular notch region, and mandibular angle. This protocol complemented other safety strategies, including control of the injection plane, preferential use of cannulas in higher-risk areas, and ultrasound guidance.

Local anesthesia was performed via mental nerve block or localized infiltration at the injection sites.

Mandibular angle marking and injection technique (male): Mandibular angle marking is considered a critical anatomical landmark (Figure 2). Posterior limit was set one fingertip anterior to the tragus, forming a triangular or L-shaped marking. The U-shaped depression, typically located 1 cm anterior to the anterior border of the masseter, was used as a guide.

The injection technique was executed using two entry points, either along the anterior border of the masseter or directly at the gonial angle, based on operator preference. A blunt 22G cannula was inserted into the subcutaneous plane, and retrograde linear injections of filler were administered (maximum 0.2 mL per line). This approach ensured uniform distribution, improved molding, and volumization with lower product demand compared to deeper planes that must overcome soft tissue and muscle thickness and to avoid deeper structures, reduce the required volume for projection, and minimize risks. Local anesthesia was limited to the cannula entry points.

Female Approach

Female aesthetic planning emphasized soft angles and refined contours. The facial shape and gonial angle were designed with subtle transitions and a comparatively smaller bigonial width to maintain delicate facial proportions. The jawline contour followed a gentle curvature, with moderate filler volumes strategically distributed to preserve femininity and harmony. Chin projection was planned with slight elongation and a tapering V-shaped configuration to achieve balance and elegance consistent with natural female aesthetics.

**Figure 3:**
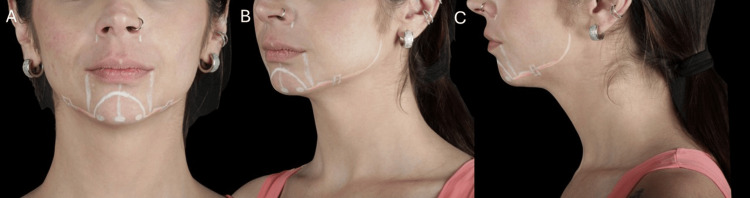
Clinical markings for chin and mandibular angle enhancement in female patients. (A) Frontal view showing the vertical alignment with oral commissures and central midline for chin projection. (B) Oblique view illustrating the distribution of three supraperiosteal injection points with central dominance to create a tapered contour. (C) Lateral view demonstrating the mandibular angle access with subcutaneous cannula approach for soft definition and contour preservation

Chin marking and injection technique (female): Chin marking followed vertical alignment with the oral commissures and a central midline (Figure 3). Typically, three injection points were marked to achieve a more refined and feminine chin contour.

The injection volume was proportioned with the central point receiving more filler. For example, if 1 mL of HA is required, the central point receives a higher volume (0.4 mL), while the lateral points receive 0.3 mL each. The injections were performed with a needle using a bolus technique in a deep supraperiosteal plane, optimizing support and structure while avoiding lateral widening. Local anesthesia involved a mental nerve block or small volumes of lidocaine infiltrations at the entry points. The approach ensured precise projection with minimal product volume, tailored to the anatomical needs of female patients.

Mandibular angle marking and injection technique (female): Female mandibular angle marking adhered to anatomical safety protocols, focusing on subtle contour definition with soft transitions rather than prominent angular projection.

**Figure 4:**
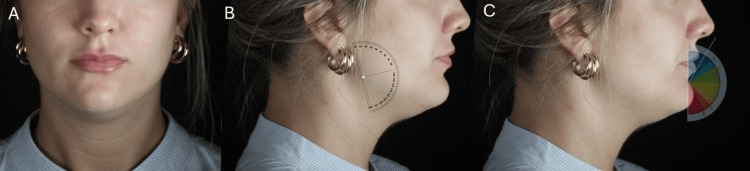
Cephalometric analysis for aesthetic planning in female patients. (A) Frontal view illustrating the bizygomatic and bigonial widths. (B) Profile view with goniometer indicating gonial angle evaluation. (C) Profile view with goniometer assessing the mentolabial angle

Cephalometric planning was based on ideal female facial proportions, including a wider bizygomatic width relative to the bigonial distance, as demonstrated in Figure 4. This evaluation supported individualized treatment decisions to preserve midface dominance while refining the lower third.

The upper access port is positioned approximately 1 cm anterior to the tragus to avoid the superficial temporal artery. The inferior port is placed 1.5 cm anterior to the anterior border of the masseter muscle, reducing the risk of injury to the facial artery and marginal mandibular nerve.

Subcutaneous injections with a 22G×50 mm blunt cannula, applying retrograde linear injections, were administered (maximum of 0.2 mL per line), gently refining contours. Local anesthesia was limited to cannula entry points.

## Results

Data were collected from 10 healthy patients aged 25-45 years old. All procedures were performed by the same injector using a standardized approach based on cephalometric analysis, anatomical landmarks, and individualized aesthetic planning. Table 1 summarizes patient demographics, main aesthetic concerns, treated areas, and filler volumes.

**Table 1:** Patient demographics, concerns, treated areas, and filler volume used

Patient ID	Age	Sex	Main concern	Filler volume (mL)	Treatment areas
1	28	M	Chin underprojection	4	Chin + prejowl
2	35	F	Mandibular contour deficiency	6	Chin + jawline
3	25	F	Chin projection	3	Chin + prejowl
4	43	F	Double chin appearance	4	Chin + jawline
5	30	M	Lack of contour	6	Chin + jawline
6	29	F	Soft chin definition	3	Chin
7	40	F	Retruded menton	5	Chin + jawline
8	33	M	Angle definition	4	Jawline + angle
9	26	F	Mandibular asymmetry	4	Chin + prejowl
10	38	M	Weak jawline	5	Chin + jawline

Consistent improvements in mandibular projection, jawline contour, and chin definition were achieved in all patients. Table 2 presents key outcomes, including pre- and post-treatment projection measurements, patient satisfaction rates, and adverse events. Patient satisfaction was high (98%), and no major adverse events were reported. Mild and transient edema and ecchymosis occurred in two cases, resolving spontaneously within 72 hours.

**Table 2:** Projection measurements, satisfaction rates, and adverse events

Patient ID	Pre-treatment projection (mm)	Post-treatment projection (mm)	Projection gain (mm)	Patient satisfaction (%)	Adverse events
1	6.5	9	2.5	100	None
2	4.1	6.6	2.5	100	None
3	4.4	6.8	2.4	100	None
4	5	8	3	100	None
5	3.2	5.1	1.9	90	None
6	5.8	8	2.2	100	None
7	5.1	7.4	2.3	100	None
8	6	8.5	2.5	100	None
9	5.5	7.3	1.8	90	None
10	4.9	6.9	2	100	None

Case study 1

A 28-year-old male patient presented with the complaint of a weak and undefined jawline, particularly in the profile view. Clinical and cephalometric analysis revealed mandibular retrognathia. His bigonial width was narrower than his bizygomatic width, contributing to a juvenilized appearance. Based on these findings, the treatment plan aimed to restore chin projection and create a more structured mandibular line.

**Figure 5:**
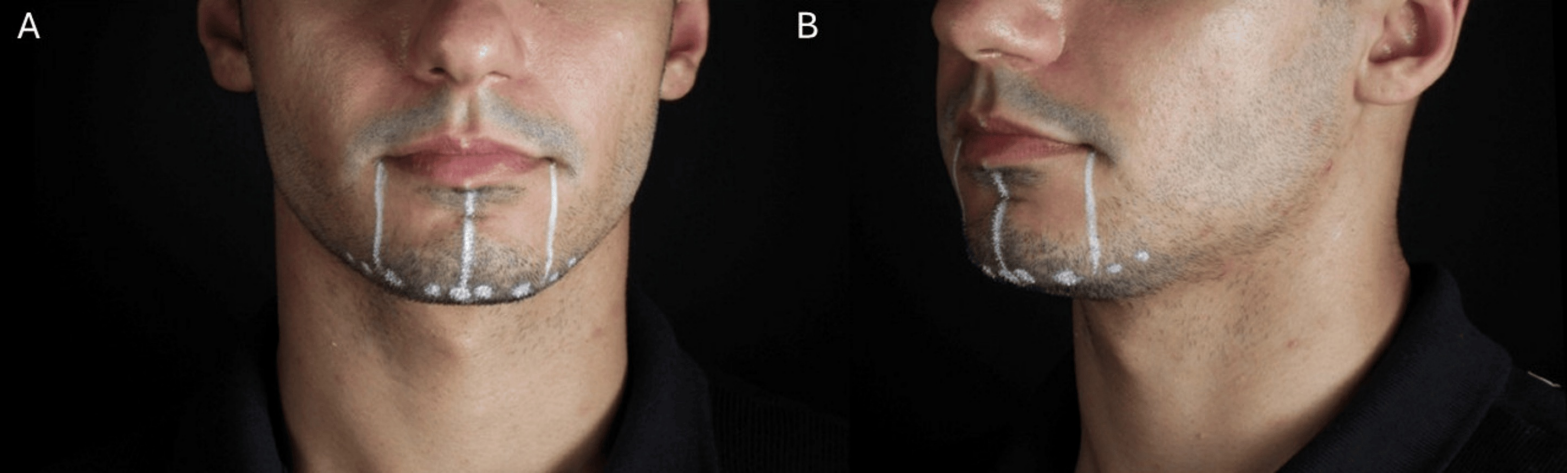
Pre-procedural markings for chin augmentation in a male patient. (A) Frontal view showing the injection points for supraperiosteal boluses. (B) Oblique view illustrating the mandibular contour alignment

**Figure 6:**
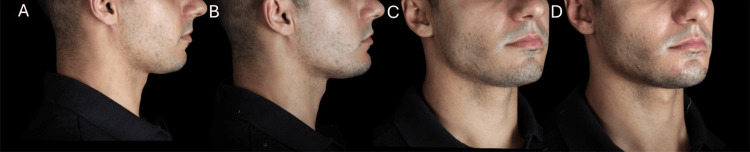
Post-treatment outcome after chin and prejowl enhancement in a male patient. (A, C) Pretreatment views. (B, D) Post-treatment views showing the improved chin projection and mandibular definition

A total of 4 mL of high G' HA filler was administered, distributed between the chin and prejowl regions, injected via supraperiosteal boluses in the chin with a needle, and along the prejowl sulcus and mandibular body using a 22G blunt cannula in the subcutaneous plane. Immediate improvement in jawline definition and chin projection was observed (Figures 5-6). Sequential lateral and oblique views demonstrated improved chin projection, enhanced definition of the mandibular line, and a more balanced lower facial contour. The treatment resulted in a sharper, more structured profile consistent with masculine aesthetic ideals.

**Figure 7:**
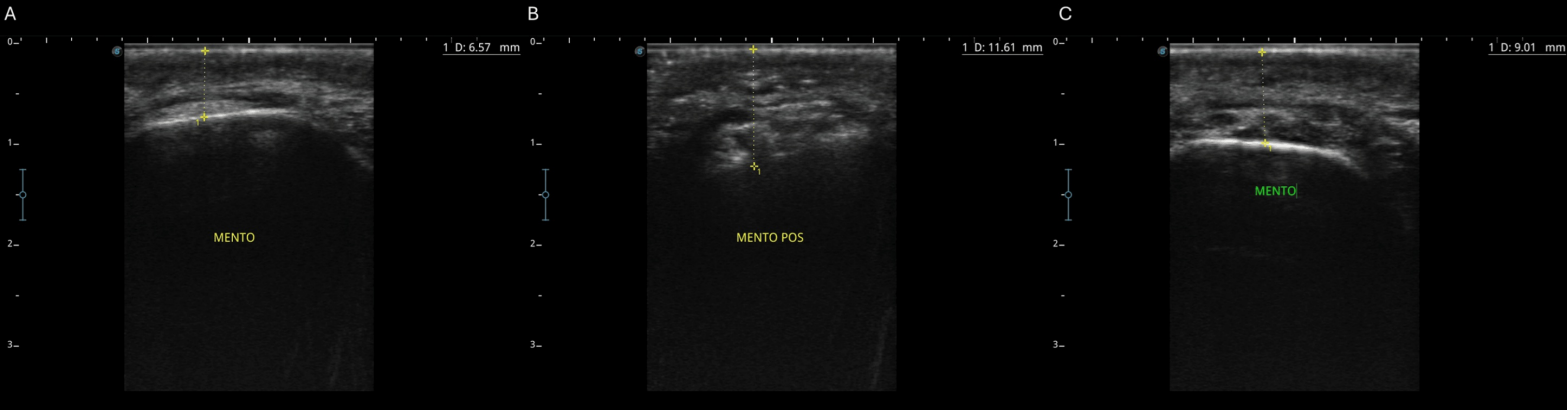
Ultrasonographic assessment of soft tissue thickness in the menton region. Sequential B-mode ultrasound images demonstrating the following: (A) baseline measurement (D0=6.57 mm), (B) immediate post-procedure thickness (POI=11.61 mm), and (C) late evaluation after 90 days (D90=9.01 mm)

Ultrasound evaluation demonstrated a baseline soft tissue thickness of 6.57 mm, increasing to 11.61 mm immediately post-procedure and stabilizing at 9.01 mm after 90 days, confirming filler integration (Figure 7).

Case study 2

A 35-year-old female patient complained of nasal overprojection in profile photographs. Upon detailed analysis, the imbalance was attributed to a retruded menton and inadequate lower facial support, rather than nasal prominence. Her profile was convex, with disproportion among facial thirds and mild jowl formation.

**Figure 8:**
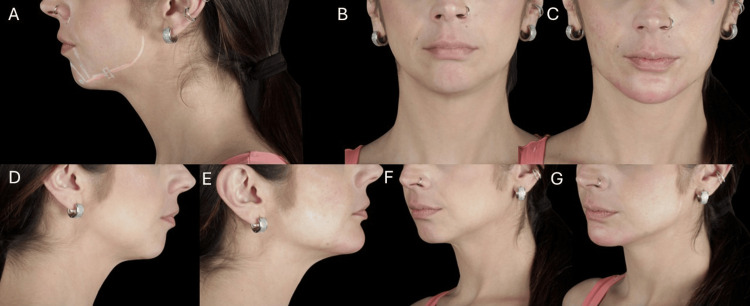
Pre- and post-treatment analysis of a female patient with chin retrusion and mandibular contour deficiency. (A) Pre-procedural facial analysis and anatomical marking. (B, C) Frontal views, before and after treatment, demonstrating the restoration of profile balance and correction of disproportion among facial thirds. (D, E) Lateral views, before and after treatment. (F, G) Oblique views, before and after treatment, illustrating the enhanced mandibular contour and harmonization of the lower third

The treatment plan focused on harmonizing the nasolabial-chin relationship and restoring contour along the mandibular line (Figure 8). A total of 6 mL of high G' HA filler was used: 2 mL were injected into the chin via three deep supraperiosteal boluses using a needle and 2 mL per side along the mandibular line and angle using a 22G×50 mm cannula in the subcutaneous plane. The intervention resulted in profile balance, improved facial thirds, and a visible enhancement in mandibular support at the 90-day follow-up (Figure 8).

Case study 3

A 25-year-old female patient presented with an aesthetic concern of insufficient horizontal chin projection, seeking subtle chin elongation without altering her well-defined mandibular angle. Cephalometric analysis revealed a mildly short vertical lower third but otherwise proportional facial architecture.

The treatment consisted of 3 mL of HA filler, with 2 mL injected into the chin using three deep supraperiosteal bolus points for structural projection, and 1 mL was distributed bilaterally across the prejowl sulcus (0.5 mL per side) via cannula.

**Figure 9:**
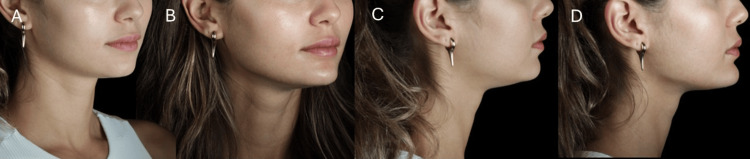
Pre- and post-treatment outcome after chin projection and prejowl contouring in a female patient with mild horizontal chin deficiency. (A, B) Oblique views, before and after treatment, showing the increased chin projection and smoother prejowl transition. (C, D) Lateral views, before and after treatment, demonstrating the elongation of the lower third and improved facial balance

**Figure 10:**
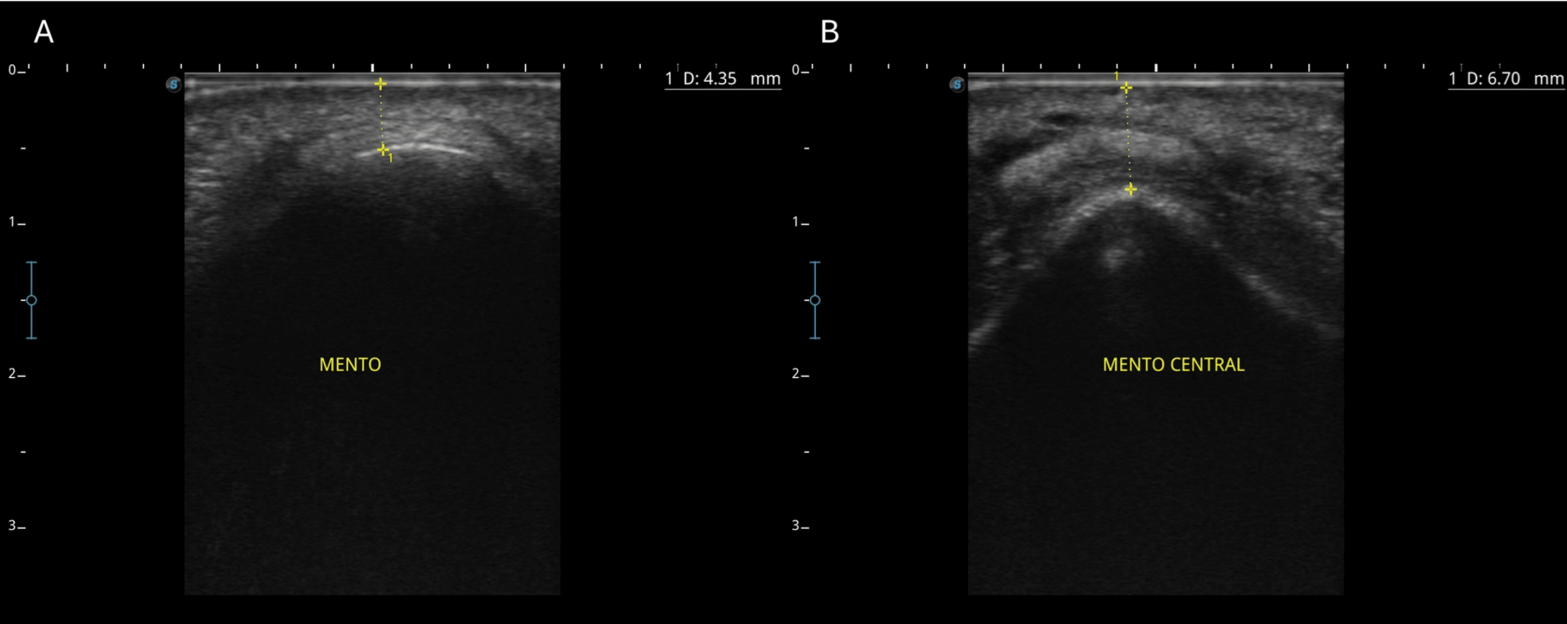
Ultrasonographic evaluation of soft tissue thickness in the menton region before and after treatment. (A) Baseline image (day 0) showing the soft tissue thickness of 4.35 mm. (B) Post-treatment evaluation at 90 days (day 90) showing the thickness of 6.70 mm, confirming a net increase of 2.35 mm and stable filler integration

Assessment at 90 days confirmed the preservation of volume and symmetry, with no adverse events reported (Figure 9). Ultrasound images showed an initial soft tissue thickness of 4.35 mm (D0) and a post-treatment measurement of 6.70 mm at 90 days (D90), confirming a net increase of 2.35 mm (Figure 10). The patient reported a more balanced appearance with no adverse effects.

Case study 4

A 43-year-old female patient was concerned about submental fullness and the appearance of a double chin. Facial analysis revealed the main contributing factor was insufficient skeletal support at the menton and lack of mandibular contour definition, contributing to ptosis of the medial fat compartment and obtuse cervicomental angle.

The treatment involved 4 mL HA filler, distributed as 1 mL in the chin (three supraperiosteal boluses with needle) and 3 mL along the mandibular contour (1.5 mL per side) via subcutaneous cannula technique.

**Figure 11:**
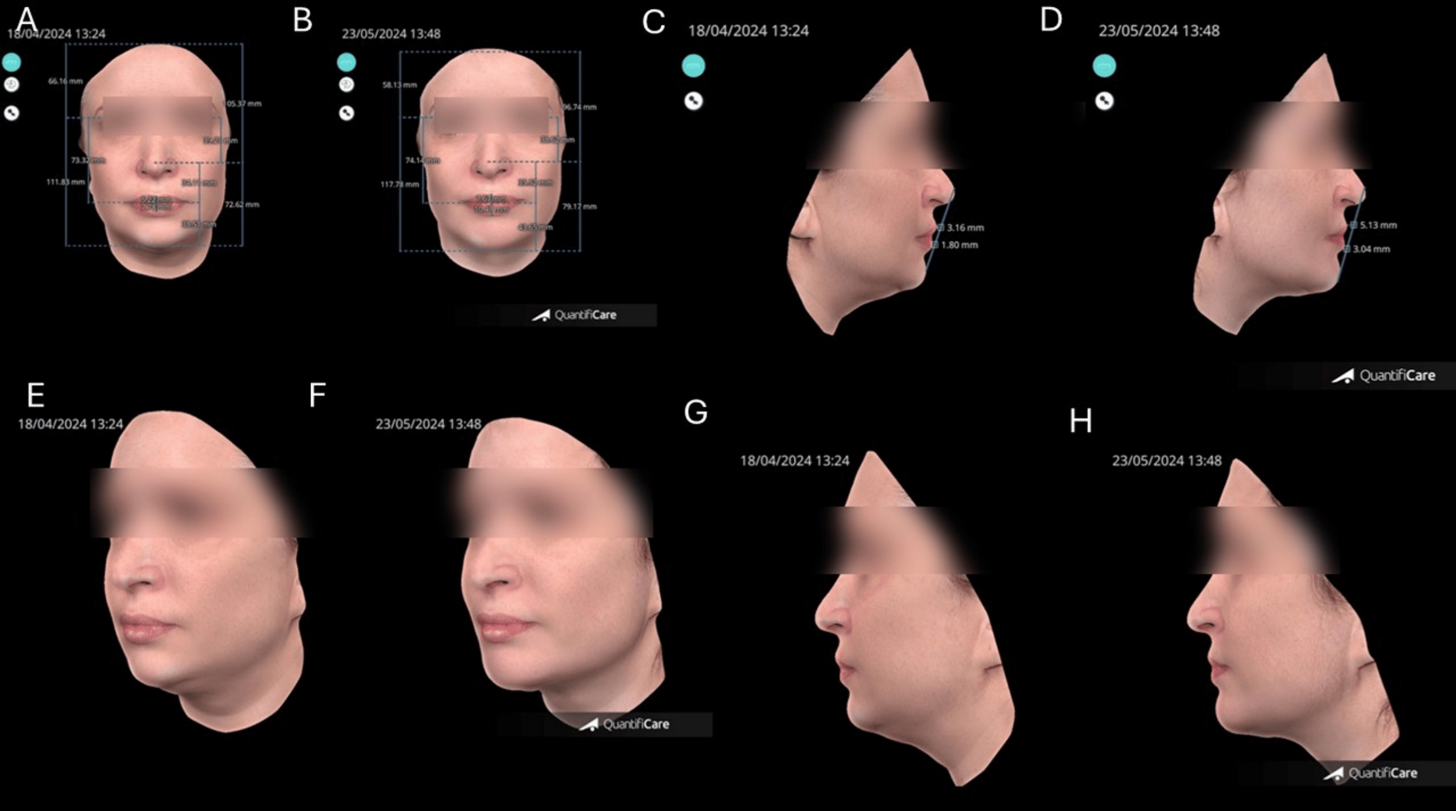
Quantitative and volumetric analysis using 3D facial scanning before and after treatment. (A, B) Comparative 3D analysis at baseline and 35 days post-treatment, showing the volumetric gain and improved mandibular projection. (C, D) Cephalometric evaluation with reference line from the menton, lower lip, and nasal tip, before and after treatment, illustrating the correction of cervicomental angle. (E, F) Oblique view, before and after treatment, highlighting the redistribution of submental fat and contour refinement. (G, H) Lateral views, before and after treatment, confirming the contour refinement Image created using QuantifiCare® (Biot, France)

The 3D image showed a notable reduction in submental volume, attributed to improved support and redistribution of lower facial fat compartments (Figure 11). Total volumetric gain in the treated zone was 4.08 mL, and the patient expressed satisfaction with the improved definition and profile.

**Figure 12:**
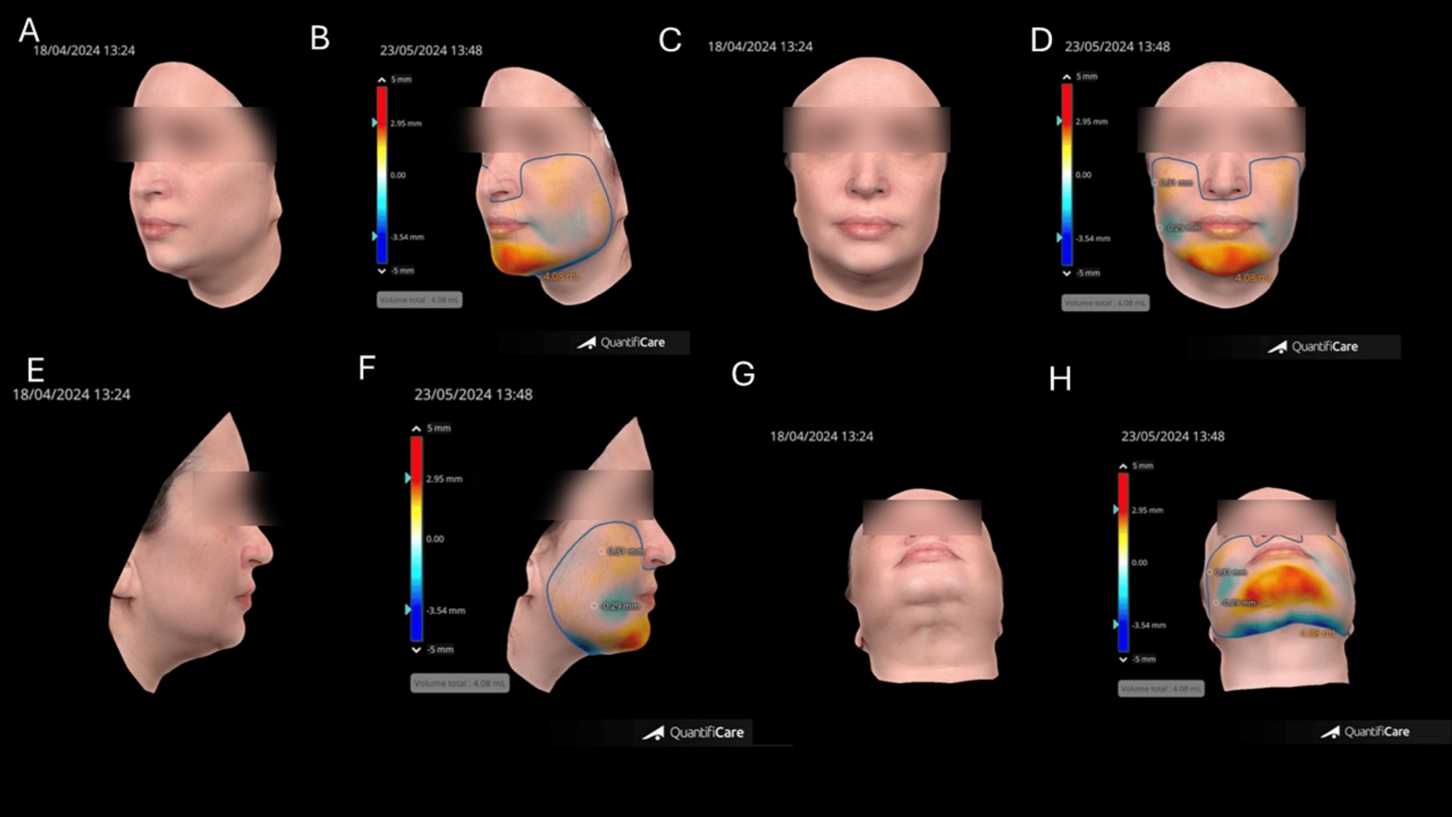
Volumetric and surface mapping analysis using 3D imaging system. Pre- and post-treatment comparison illustrating the topographic volume gain (total: 4.08 mL) in the chin and mandibular contour area. Color-coded heatmaps showing the increased projection and soft tissue displacement in key regions (red/yellow), confirming targeted volume distribution and structural support. (A, B) Oblique views, before and after treatment. (C, D) Frontal views, before and after treatment. (E, F) Lateral views, before and after treatment. (G, H) Focused submental views, before and after treatment Image created using QuantifiCare® (Biot, France)

The patient reported high satisfaction, and no adverse events were recorded. The outcome aligns with findings in the literature emphasizing the relevance of deep structural projection in optimizing soft tissue support and contour harmony [7]. The data confirm the volumetric effectiveness and structural support achieved through targeted filler application (Figure 12).

Case study 5

A 30-year-old male patient with a history of orthodontic correction for retrognathia reported dissatisfaction with his "childlike" and underdeveloped facial appearance. Facial hair was previously used to camouflage mandibular deficiency.

Analysis showed an inverted proportion between the midface and lower third (bizygomatic width greater than bigonial width), marked chin retrusion, and a very thin subcutaneous layer, which facilitated product projection.

It is clinically relevant to note that many male patients grow facial hair to compensate for weak chin projection or undefined mandibular borders, often masking the extent of volume deficiency [16].

A customized plan utilized a total of 6 mL of filler, with 2 mL in the chin (six supraperiosteal boluses) and 2 mL per side in the mandibular angle through access points 1 and 2 using a 22G blunt cannula in the subcutaneous plane.

Of note, this patient exhibited the thinnest soft tissue layer among the cases evaluated, which favored product projection and visual efficacy. Despite the pronounced skeletal deficiency, the reduced skin thickness allowed for superior contour definition and aesthetic improvement, as documented in literature discussing tissue thickness as a determinant of filler performance [3,4,17].

**Figure 13:**
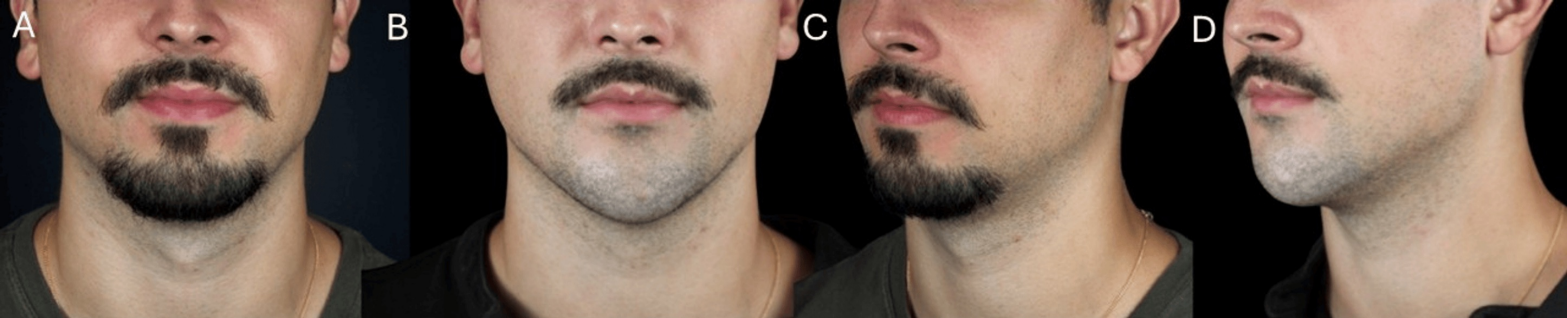
Post-treatment result in a male patient with retrognathia after chin and mandibular angle enhancement. (A, B) Frontal views, before and after treatment. (C, D) Oblique views, before and after treatment

**Figure 14:**
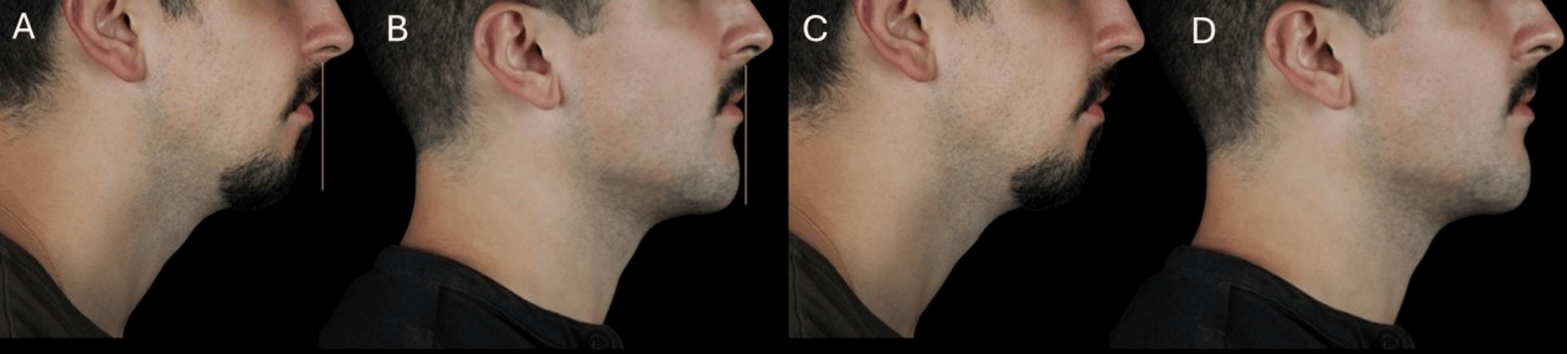
Lateral view comparison before and after chin and mandibular angle enhancement in a male patient with retrognathia. (A, B) Right profile views, before and after treatment, with reference line for chin projection comparison. (C, D) Left profile views, before and after treatment

The treatment resulted in enhanced chin projection, restoration of mandibular angle volume, and an overall transformation toward a more structured and masculine appearance at the 90-day follow-up (Figures 13-14), with no adverse events and excellent patient-reported outcomes.

## Discussion

The clinical cases presented demonstrate the versatility, efficacy, and aesthetic value of using HA fillers in chin and mandibular contour enhancement. Through individualized treatment planning based on anatomical, cephalometric, and morphological assessments, it is possible to achieve natural and harmonious results in both male and female patients, not only addressing volumetric deficiencies but also enhancing facial balance and structural support.

The use of deep supraperiosteal and subcutaneous techniques, guided by anatomical knowledge and tailored to soft tissue characteristics, proved effective in optimizing projection and contour with minimal product volumes. Additionally, diagnostic tools such as ultrasonography and 3D facial imaging enabled the objective evaluation of outcomes, reinforcing the reproducibility and predictability of this approach.

These findings highlight the importance of a comprehensive facial assessment that extends beyond the initial area of complaint, including the identification of underlying skeletal discrepancies that may influence the perception of disharmony. Addressing these foundational elements leads to more profound and satisfying aesthetic results.

Future studies with larger sample sizes and long-term follow-up will further validate the impact of this method on facial aesthetics, soft tissue behavior, and patient satisfaction, consolidating its role as a cornerstone in minimally invasive lower face enhancement.

The lower third of the face plays a critical role in perceived facial aesthetics, directly influencing perceptions of youth, attractiveness, and gender-specific characteristics and identity. Anatomical deficiencies such as mandibular hypoplasia, obtuse gonial angles, and retrogenia can significantly alter facial proportions, leading to aesthetic disharmony and functional concerns. Non-surgical mandibular reshaping with HA fillers has emerged as a safe and effective technique to address these concerns, offering immediate and natural-looking results with minimal downtime [5,18].

The technique described in this study emphasizes individualized aesthetic planning based on anatomy, using high G’ HA fillers to restore support and projection in a targeted, volume-efficient manner, resulting in aesthetic refinement. This is consistent with findings by Muhn et al., who highlighted that HA fillers with high elastic modulus provide better tissue lift and long-term shape retention, particularly in structurally demanding areas such as the chin and jawline [18].

When executed with a proper understanding of anatomical structures and respecting danger zones, the risks of complications remain low. This aligns with the guidelines discussed by Wollina and Goldman, which emphasize the importance of respecting vascular and neural landmarks, such as the facial artery, mental foramen, and marginal mandibular nerve, to reduce the risk of vascular occlusion or neurosensory complications. In the technique presented, both deep supraperiosteal boluses and subcutaneous linear threads were performed in accordance with these safety principles, ensuring filler deposition away from high-risk zones [17].

Gender-specific cephalometric planning was incorporated in the treatment algorithm. As supported by Mommaerts, the ideal male mandibular angle typically ranges between 90° and 110°, promoting a squarer and more robust profile, whereas in females, softer and more obtuse angles are aesthetically preferred. The planning in this protocol accounted for cephalometric harmony by evaluating bigonial-to-bizygomatic proportions and adapting filler distribution accordingly [19].

The effectiveness of this technique is also evidenced by high patient satisfaction, as observed in previous case reports such as that by Arora et al., in which non-surgical lower face contouring significantly improved mandibular definition with minimal adverse effects [20]. The use of only 2-6 mL of filler in total demonstrates an optimized product use, particularly when compared to higher volumes often required in deep plane augmentation [21].

This study has some limitations that should be acknowledged. It is a prospective case series conducted in a routine clinical practice setting, with a small sample size that may not represent the broader population and follow-up limited to early- and mid-term assessments (up to 90 days), which does not allow definitive conclusions about the durability of outcomes. To more adequately assess long-term durability, future studies should include follow-up periods of at least 6-12 months. In addition, cephalometric ratios and golden ratio parameters were used qualitatively to guide individualized planning and were not collected as standardized quantitative endpoints for all cases.

Importantly, the integration of diagnostic ultrasound imaging during and after the procedure further enhances the safety profile by confirming injection plane accuracy and monitoring for potential complications, aligning with contemporary safety standards in aesthetic practice.

Anatomically guided mandibular contouring using HA fillers is a scientifically supported, safe, and highly adaptable technique. When tailored to individual morphology and executed with precise control over anatomical planes and filler properties, it offers reproducible, natural results with a low complication profile. This approach respects both structural dynamics and aesthetic harmony, reinforcing its role in contemporary facial rejuvenation strategies.

## Conclusions

Our findings suggest that non-surgical chin and jawline enhancement with a high G′ HA filler can provide natural-looking improvements using relatively small product volumes when guided by anatomical and cephalometric planning, yet acknowledging the limitations of this small, single-center case series. The protocol described, based on supraperiosteal and subcutaneous injection planes, gender-specific aesthetic parameters, and ultrasound guidance, was associated with high patient satisfaction and no major adverse events in the cases presented.

## References

[1] Kaya KS, Türk B, Cankaya M, Seyhun N, Coşkun BU (2019). Assessment of facial analysis measurements by golden proportion. Braz J Otorhinolaryngol.

[2] Mendelson B, Wong CH (2012). Changes in the facial skeleton with aging: implications and clinical applications in facial rejuvenation. Aesthetic Plast Surg.

[3] Sykes JM, Suárez GA, Trevidic P, Cotofana S, Moon HJ (10.1016/B978-0-3201823-35876-7.00002-9). Applied facial anatomy. Master Techniques in Facial Rejuvenation.

[4] Go BC, Frost AS, Friedman O (2023). Using injectable fillers for chin and jawline rejuvenation. World J Otorhinolaryngol Head Neck Surg.

[5] Moradi A, Shirazi A, David R (2019). Nonsurgical chin and jawline augmentation using calcium hydroxylapatite and hyaluronic acid fillers. Facial Plast Surg.

[6] Vanaman Wilson MJ, Jones IT, Butterwick K, Fabi SG (2018). Role of nonsurgical chin augmentation in full face rejuvenation: a review and our experience. Dermatol Surg.

[7] Rauso R, Rugge L, Chirico F (2022). Nonsurgical reshaping of the lower jaw with hyaluronic acid fillers: a retrospective case series. Dermatol Pract Concept.

[8] Kim JE, Sykes JM (2011). Hyaluronic acid fillers: history and overview. Facial Plast Surg.

[9] Salwowska NM, Bebenek KA, Żądło DA, Wcisło-Dziadecka DL (2016). Physiochemical properties and application of hyaluronic acid: a systematic review. J Cosmet Dermatol.

[10] Pallett PM, Link S, Lee K (2010). New "golden" ratios for facial beauty. Vision Res.

[11] Velemínská J, Jaklová LK, Kočandrlová K, Hoffmannová E, Koudelová J, Suchá B, Dupej J (2022). Three-dimensional analysis of modeled facial aging and sexual dimorphism from juvenile to elderly age. Sci Rep.

[12] Hajeer MY, Mao Z, Millett DT, Ayoub AF, Siebert JP (2005). A new three-dimensional method of assessing facial volumetric changes after orthognathic treatment. Cleft Palate Craniofac J.

[13] Marolt C, Freed B, Coker C (2021). Key anatomical clarifications for the marginal mandibular branch of the facial nerve: clinical significance for the plastic surgeon. Aesthet Surg J.

[14] Constanza AK, Marta AM, Ignacio NC (2022). Facial artery, an essential anatomy in different specialties: a review. J Otolaryngol ENT Res.

[15] Clark NW, Pan DR, Barrett DM (2023). Facial fillers: relevant anatomy, injection techniques, and complications. World J Otorhinolaryngol Head Neck Surg.

[16] Braz A, Eduardo CC (2020). The facial shapes in planning the treatment with injectable fillers. Indian J Plast Surg.

[17] Wollina U, Goldman A (2020). Facial vascular danger zones for filler injections. Dermatol Ther.

[18] Muhn C, Rosen N, Solish N (2012). The evolving role of hyaluronic acid fillers for facial volume restoration and contouring: a Canadian overview. Clin Cosmet Investig Dermatol.

[19] Mommaerts MY (2016). The ideal male jaw angle - an internet survey. J Craniomaxillofac Surg.

[20] Arora RT, Arora S, Kaushik I, Patil C (2023). Non-surgical lower face contouring in an Indian patient: a case study. Cureus.

[21] Peng JH, Peng HP (2024). Treating the double chin with hyaluronic acid injection. J Cosmet Dermatol.

